# Computer Games and Prosocial Behaviour

**DOI:** 10.1371/journal.pone.0094099

**Published:** 2014-04-09

**Authors:** Friederike Mengel

**Affiliations:** 1 Department of Economics, University of Essex, Wivenhoe Park, Colchester, Essex, United Kingdom; 2 Department of Economics (AE 1), Maastricht University, MD Maastricht, Limburg, The Netherlands; Institutes for Behavior Resources and Johns Hopkins University School of Medicine, United States of America

## Abstract

We relate different self-reported measures of computer use to individuals' propensity to cooperate in the Prisoner's dilemma. The average cooperation rate is positively related to the self-reported amount participants spend playing computer games. None of the other computer time use variables (including time spent on social media, browsing internet, working etc.) are significantly related to cooperation rates.

## Introduction

There is widespread concern about the effects of increased time spent playing computer games on social behaviour of adolescents. In this note we study the relationship between the time undergraduate students spend playing computer games with what is probably the most common measure of prosocial behaviour in lab experiments, namely an individual's propensity to cooperate in the Prisoner's dilemma.

There are several existing studies surveying people who spend a lot of their time playing computer games. The literature has identified a variety of links between game playing and social behaviour. It has consistently pointed to a positive link between playing violent computer games and aggression, but has remained less conclusive as to whether game playing *per se* is associated with less prosocial behaviour. On the negative side playing (violent) computer games or playing excessively has been associated with increased aggression ([1]; [2]; [3]; [4]; [5]), increased anxiety ([6]), depression ([7]), less prosocial behaviour ([8]; [5]) and lower self-esteem in women ([9]). The study by [5] differs from much of the literature in that it attempts to draw a causal link between exposure to violent video games and increased aggressive behaviour, aggressive cognition and affect and decreased empathy and prosocial behavior. They conduct a meta-analytic review on longitudinal studies which includes cross-cultural comparisons between eastern and western countries and do find support for such causal links.

[10] summarize the research on the benefits of playing video games in terms of cognitive, motivational, emotional and social factors. Players certainly seem to acquire important prosocial skills when they play games specifically designed to reward cooperation. In fact, evidence from several correlational and longitudinal studies suggests that playing prosocial video games relates to prosocial behaviours (see e.g. [11] or [12]). More specifically, [11] found that playing pro-social games led to causal, short-term effects on “helping” behaviours and longitudinal effects were also found, in that children who played more prosocial games at the beginning of the school year were more likely to exhibit helpful behaviors later that year. In a large-scale representative U.S. study [13] showed that adolescents who played games with civic experiences were more likely to be engaged in social and civic movements in their everyday lives (e.g. raising money for charity, volunteering and persuading others to vote). [14] have found a positive relation between game playing and variables such as family closeness, activity involvement, positive school engagement, positive mental health, self-concept, friendship network, and obedience to parents. To sum up, the literature has shown that violent, non-cooperative games tend to associated with less pro-social behaviour, while games with a pro-social content tend to be associated with more pro-social behaviour. Less is known about how computer use patterns and in particular the time spent playing per se, irrespective of the content of the game, affects pro-social behaviour.

Our study differs both methodologically and conceptually from this literature. While we conduct a controlled laboratory experiment with an incentivized measure of prosocial behavior, the existing literature consists mostly of survey studies or experimental designs where behaviour immediately after playing violent computer games is observed. The existing literature also usually focuses on violent games ([5]; [4]; [9]) or on excessive playing of computer games ([8]; [15]), though there is some literature on pro-social games as well ([11]). By contrast, in our study we consider any computer games and we do not select a particular sample of game players. The randomly selected participants in our study play between 0 to 8 hours per day.

We measure prosocial behaviour among a sample of undergraduate students using the prisoner's dilemma game. In this game people simultaneously choose between two actions: cooperate or defect. If both players cooperate they receive the jointly efficient cooperation payoff (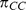). If both defect they receive the inefficient payoff (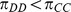). However, if one player defects and the other cooperates the defector receives the highest possible payoff, the “temptation” payoff 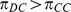, while the cooperator receives the lowest possible payoff, the “sucker payoff” 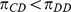.

## Methods

We asked undergraduate students at the University of Essex (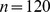) to play the Prisoner's dilemma game for 10 periods. Participants volunteered for the experiment by signing up online. They were recruited using the recruitment software hroot and provided written consent to participate in the experiment. At the time of registering they did not know that the experiment was about the prisoner's dilemma game or would include questions about computer use. This is standard procedure in Economics and at EssexLab at the University of Essex and has been approved by the University ethics procedures and the University of Essex Faculty Ethics committee.

In each period participants were randomly and anonymously rematched to another participant. At the end of the 10 periods they filled in a questionnaire that, apart from demographics, contained five questions about their computer habits: how much time per day they spend on the computer in total and how much of that (i) for work, (ii) using social media, (iii) playing computer games and (iv) browsing the internet. Table S1 in File S1 shows descriptive statistics regarding these and other variables. The data used in this paper are available from my webpage https://sites.google.com/site/friederikemengel/.

## Results

**Figure 1:**
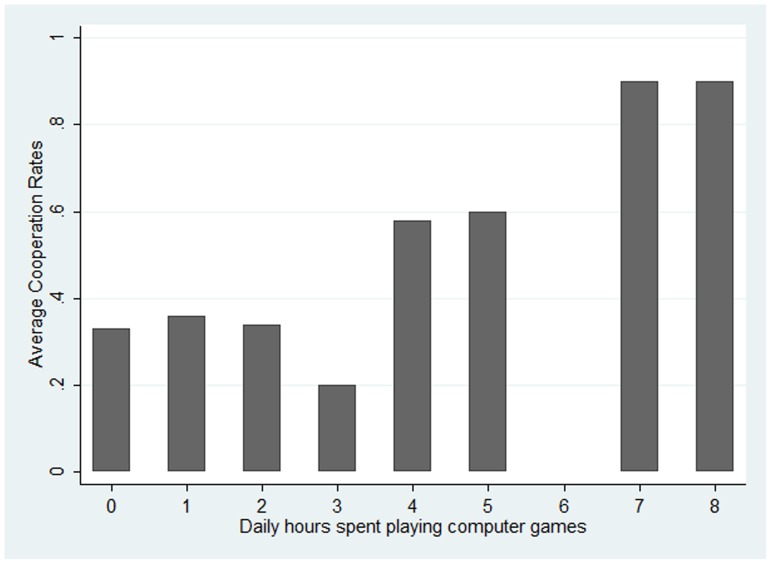
Average Cooperation Rates by hours/day spent playing computer games.

**Figure 2:**
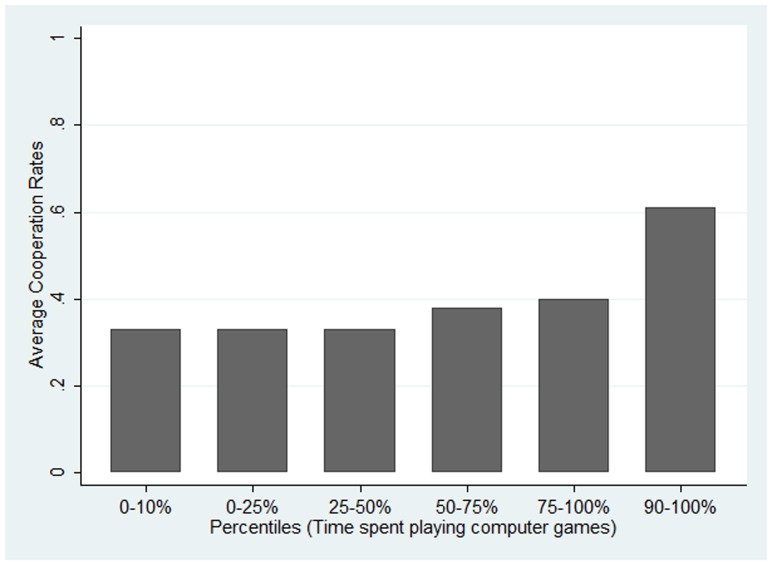
Average Cooperation Rates for different quartiles (as well as lowest and highest 10 percent) of the distribution of time spent playing computer games.

We then ask whether average cooperation rates differ for those spending more time playing computer games compared to those spending less. Figure 1 shows average cooperation rates for participants spending 0,…,8 hours per day playing computer games. On average people cooperate around 35 percent of the time across the ten periods. This is in line with existing literature ([16]; [17] [18]; [19]; [20]). Average cooperation rates are *higher* among those spending *more* time playing computer games. They exceed 50 percent for those spending more than 4 hours on the computer and even reach 90 percent for those playing 7 or 8 hours per day. The Pearson correlation coefficient between time spent playing computer games and percent choice to cooperate was 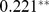 (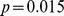). Since most of our participants spend two hours or less per day playing computer games, we also illustrate the cooperation rates for the different percentiles of the distribution (Figure 2). It can be seen that those in the highest two quartiles and in particular in the highest decile cooperate more often than others.

Table 1 addresses the statistical significance of these findings. The endogenous variable is the average rate of cooperation across all 10 periods. The exogenous variables are the time spent on the computer (i) overall (total time), (ii) for work (time work), (iii) on social media (time social media), (iv) playing computer games (time games) and (v) browsing the internet (time browsing) as well as age and a dummy which takes the value one for women. In column (1) we regress on all computer-use variables controlling for age and gender, in column (2) we omit these controls (age and gender) and in column (3) we omit all jointly insignificant computer use variables with the exception of total time. The coefficient on time games is the only significant coefficient in all three columns and the value of the coefficient is about 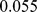 throughout. Hence one additional hour spent playing computer games increases the average cooperation rate by about 5 percentage points in our sample.

**Table 1:** OLS regression of average cooperation rate on computer use variables and demographics.

	(1)	(2)	(3)
total time (hrs)	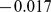	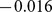	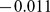
	(0.012)	(0.012)	(0.007)
time work	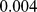	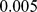	
	(0.015)	(0.015)	
time social media	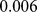	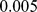	
	(0.015)	(0.014)	
time games	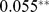	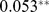	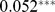
	(0.021)	(0.021)	(0.019)
time browsing	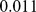	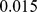	
	(0.026)	(0.025)	
age	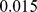		
	(0.017)		
female	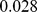		
	(0.058)		
constant	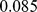	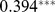	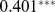
	(0.358)	(0.064)	(0.062)
Observations	120	120	120
R2	0.077	0.066	0.065

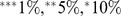
 significance.

Table 1 has focused on the average cooperation rate across all ten periods. One might ask whether there are differential effects with respect to the period of play. Computer game players might e.g. be more cooperative initially but not in later periods or vice versa or they might be more cooperative across all periods. Different effects could point to different underlying motives. If for example, computer game players only cooperate more in late periods, then it might be the case that they are simply less strategic than others in the sense that they are worse at anticipating the so-called “endgame effect” ([16]). If they always cooperate more than then this suggests that they are more pro-socially inclined.

Table 2, hence, shows results of random effects regressions, where the time structure is taken into account. In these regressions the endogenous variable is a binary variable indicating whether a participant cooperated in a certain period. The exogenous variables are the “computer time” variables as well as a variable indicating the period 1,…,10. Columns (1) and (2) include interactions between period and all “computer time” variables (i.e. 5 interaction terms), while columns (3) and (4) do not. The results show that participants cooperate less over time (negative coefficient on period), which is a typical result ([16]; [17] [18]; [19]). They also show that participants who spend more time playing computer games cooperate more. The interaction effect between time games and period included in columns (1) and (2) is insignificant. Computer game players cooperate more irrespective of the period of play. There is one marginally significant interaction term in columns (1) and (2) and it refers to the interaction between period and total time spent on the computer. The coefficient on the interaction term is positive (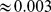), implying that the marginally negative effect observed disappears in the final periods of play. None of the other computer time variables are significant.

**Table 2:** Random Effects OLS regressions regress the binary variable indicating cooperation on time and computer use variables (VCE robust standard errors).

	(1)	(2)	(3)	(4)
total time (hrs)	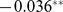	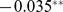	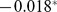	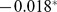
	(0.011)	(0.015)	(0.010)	(0.010)
time work	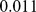	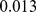	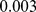	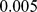
	(0.019)	(0.020)	(0.014)	(0.015)
time social media	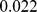	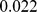	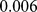	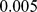
	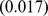	(0.018)	(0.013)	(0.013)
time games	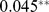	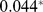	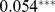	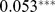
	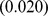	(0.024)	(0.019)	(0.019)
time browsing	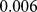	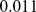	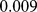	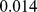
	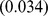	(0.034)	(0.025)	(0.025)
Period	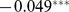	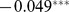	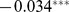	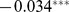
	(0.011)	(0.011)	(0.004)	(0.004)
age	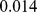		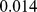	
	(0.014)		(0.014)	
female	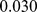		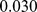	
	(0.063)		(0.063)	
constant	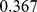	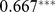	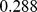	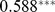
	(0.296)	(0.064)	(0.292)	
Observations	1200	1200	1200	1200
Period Interactions	YES 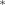	YES 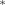	NO	NO
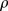	0.332	0.329	0.332	0.329

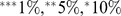
 significance.

## Discussion

We conclude that participants in our study who spent more time playing computer games display more prosocial behavior. It is important to note that we cannot make any claims regarding causality. It is both possible, given our results, that more prosocial people self-select into playing more computer games as it is possible that playing computer games “makes” people more prosocial.

While the effects we find are highly statistically significant and the effect size considerable (a 5 percentage points increase in the frequency of cooperation per additional hour spent playing computer games), the computer use variables elicited in this study account for less than 8 percent of the variation in behaviour (see Table 1). Typical factors that account for much of the variation in average cooperation rates in a 10-period repeated prisoner's dilemma are the “history of play” which includes the behaviour of the opponent and the payoff parameters (see e.g. [17] or [19]).

Interestingly, we find no effects of other computer use variables, such as time spent browsing or working or time spent on social media sites. With respect to the latter, recent studies have suggested a negative association between the use of social media and empathy (see e.g. [21]). In terms of behaviour in the prisoner's dilemma we find no relation between social media use and pro-sociality.

The positive association between computer game playing and prosocial behaviour is found in a sample of undergraduate students spending between 0 and 8 hours per day playing computer games, with the great majority playing four hours or less. This contrasts with much of the literature where samples of necessarily pathological, excessive or addicted game players are considered. For such samples different effects have been documented in the literature. Also mostly negative effects have been established when focusing on violent games (e.g. [8] or [5]), though not exclusively. At least in the short run, positive relationships have been documented e.g. by [22] or [23] if the game, albeit being violent, contains elements of cooperative nature. Our study does allow us to conclude, however, that extensive playing of computer games is not always associated with more antisocial behaviour. It also suggests that out of a number of variables describing computer usage, time spent playing computer games might be the most important in understanding the relationship between pro-sociality and computer usage.

Future research is needed to understand how robust the effect is when considering different samples and different measures of pro-sociality. Future research is also needed to get some insight into the crucial issue of causality and to gain insight into the mechanisms at work behind these mere correlations. Understanding the mechanisms might help shed light into why different studies on this subject have sometimes come to such different conclusions.

## Supporting Information

File S1 Variables. This file contains Table S1. Table S1. Summary statistics of variables used in regression.
(PDF)

File S2 Experimental Instructions.
(PDF)
