# Supplementary material for: Computer Games and Prosocial Behaviour
Source: PLoS One. 2014 Apr 9;9(4):e94099. doi: 10.1371/journal.pone.0094099 (PMC3981774; doi:10.1371/journal.pone.0094099)
Supplement: File S1 — Variables. This file contains Table S1. Table S1. Summary statistics of variables used in regression. (PDF) [file pone.0094099.s001.pdf]

Computer Games and prosocial behaviour - Supplementary Material S1

Friederike Mengel [*,†]

University of Essex & Maastricht University

March 15, 2014

S1: Variables

Table S1 shows summary statistics regarding all the variables used in regression (1) in the main manuscript. Additional summary statistics of our 120 participants is available that we cannot relate to individual decisions. 43 participants were from the UK and 45 not from the UK. 62 participants were christian, 37 not religious, 4 muslims and 2 buddhists. 80 participants were of white origin, 10 chinese, 8 black african, 4 black carribean, 4 south asian and 3 mixed. Numbers that are missing to 120 were non-respondents on the respective questions.

	Description	Range	Mean	1st Quartile	3rd Quartile
average cooperation	Average Cooperation Rate across 10 periods	[0,1]	0.35	0.1	0.5
total time (hrs)	Time spent on computer in hrs per day	[1,18]	7.76	5	10
time work	Time spent working on computer in hrs/day	[0,10]	3.37	2	4
time social media	Time spent on social media in hrs/day	[0,12]	2.57	1	3
time games	Time spent playing computer games hrs/day	[0,8]	1.38	0	3
time browsing	Time spent browsing internet hrs/day	[0,5]	1.61	1	2
period	period	[1,10]	5.5	3	8
age	age	[18,28]	20.35	18	21
female	takes value 1 for women and 0 for men	[0,1]	0.61	0	1

Table S1: Summary statistics of variables used in regression.

[*] Department of Economics, University of Essex, Wivenhoe Park, Colchester CO4 3SQ, Essex, United Kingdom. *e-mail*: fr.mengel@gmail.com

[†] Department of Economics (AE 1), Maastricht University, PO Box 616, 6200 MD Maastricht, Limburg, The Netherlands. *e-mail*: fr.mengel@gmail.com
