# Supplementary material for: Computer Games and Prosocial Behaviour
Source: PLoS One. 2014 Apr 9;9(4):e94099. doi: 10.1371/journal.pone.0094099 (PMC3981774; doi:10.1371/journal.pone.0094099)
Supplement: File S2 — Experimental Instructions. (PDF) [file pone.0094099.s002.pdf]

Computer Games and prosocial behaviour - Supplementary Material S2

FRIEDERIKE MENGEL *,†
University of Essex & Maastricht University

March 15, 2014

S2: Experimental Instructions

Welcome and thanks for participating at this experiment. Please read these instructions carefully. They are identical for all the participants with whom you will interact during this experiment.

If you have any questions please raise your hand. One of the experimenters will come to you and answer your questions. From now on communication with other participants is not allowed. If you do not conform to these rules we are sorry to have to exclude you from the experiment. Please do also switch off your mobile phone at this moment.

For your participation you will receive 2 pounds. During the experiment you can earn more. How much depends on your behavior and the behavior of the other participants. During the experiment we will use ECU (Experimental Currency Units) and at the end we will pay you in pounds according to the exchange rate 1 pound = 300 ECU. All your decisions will be treated confidentially.

THE EXPERIMENT

The experiment consists of 10 periods. At the start of each period you will be randomly matched with another participant. You will then in each period play the following game with the participant you were matched with in that period.

The other participant chooses		
	A	B
You choose A	a,a	b,c
You choose B	c,b	d,d

In the table your actions and payoffs are given in dark grey and your neighbours actions and payoffs in light grey. The table is read as follows (dark payoffs):

- If you choose A and the other participant chooses A, you receive a

*Department of Economics, University of Essex, Wivenhoe Park, Colchester CO4 3SQ, Essex, United Kingdom. *e-mail*: fr.mengel@gmail.com

†Department of Economics (AE 1), Maastricht University, PO Box 616, 6200 MD Maastricht, Limburg, The Netherlands. *e-mail*: fr.mengel@gmail.com

- If you choose A and the other participant chooses B, you receive b
- If you choose B and the other participant chooses A, you receive c
- If you choose B and the other participant chooses B, you receive d

Note that the other participant (light payoffs) is in the same situation as you are. This means that for the other participant:

- If the other participant chooses A and you A, the other participant receives a
- If the other participant chooses A and you B, the other participant receives b
- If the other participant chooses B and you A, the other participant receives c
- If the other participant chooses B and you B, the other participant receives d

// [**NOTE: Payoffs used in the experiment were** $(a, b, c, d) = (400, 100, 450, 120)$ **and** $(a, b, c, d) = (400, 100, 450, 200)$] **with equal frequency across sessions.//**

At the end of each period you will be informed of your choice, the choice of the other participant and your payoffs in that round.

Keep in mind that the other participant you are matched with changes in each period.

After the last period has been completed you will be asked to fill in a short questionnaire.

Your earnings in the experiment will be the sum of payoffs obtained in each round (exchanged into pounds according to the exchange rate above) plus the 2 pound show up fee.

Enjoy the Experiment!
